# Predicting the burden of cancer in Switzerland up to 2025

**DOI:** 10.1371/journal.pgph.0001112

**Published:** 2022-10-14

**Authors:** Bastien Trächsel, Elisabetta Rapiti, Anita Feller, Valentin Rousson, Isabella Locatelli, Jean-Luc Bulliard

**Affiliations:** 1 Center for Primary Care and Public Health (Unisanté), University of Lausanne, Lausanne, Switzerland; 2 Geneva Cancer Registry, University of Geneva, Geneva, Switzerland; 3 Foundation National Institute for Cancer Epidemiology and Registration (NICER), Zurich, Switzerland; 4 National Agency for Cancer Registration (NACR) Operated by NICER, Zurich, Switzerland; Babcock University, NIGERIA

## Abstract

Predicting the short-term evolution of the number of cancers is essential for planning investments and allocating health resources. The objective of this study was to predict the numbers of cancer cases and of the 12 most frequent cancer sites, and their age-standardized incidence rates, for the years 2019–2025 in Switzerland. Projections of the number of malignant cancer cases were obtained by combining data from two sources: forecasts of national age-standardized cancer incidence rates and population projections from the Swiss Federal Statistical Office. Age-standardized cancer incidence rates, approximating the individual cancer risk, were predicted by a low-order Autoregressive Integrated Moving Average (ARIMA) model. The contributions of changes in cancer risk (epidemiological component) and population aging and growth (demographic components) to the projected number of new cancer cases were each quantified. Between 2018 and 2025, age-standardized cancer incidence rates are predicted to stabilize for men and women at around 426 and 328/100,000, respectively (<1% change). These projected trends are expected for most cancer sites. The annual number of cancers is expected to increase from 45,676 to 52,552 (+15%), more so for men (+18%) than for women (+11%). These increases are almost entirely due to projected changes in population age structure (+12% for men and +6% for women) and population growth (+6% for both sexes). The rise in numbers of expected cancers for each site is forecast to range from 4.15% (thyroid in men) to 26% (bladder in men). While ranking of the three most frequent cancers will remain unchanged for men (1^st^ prostate, 2^nd^ lung, 3^rd^ colon-rectum), colorectal cancer will overtake by 2025 lung cancer as the second most common female cancer in Switzerland, behind breast cancer. Effective and sustained prevention measures, as well as infrastructural interventions, are required to counter the increase in cancer cases and prevent any potential shortage of professionals in cancer care delivery.

## Introduction

Predicting the future burden of cancer in a country has a pivotal role in cancer control planning. It provides evidence for policymakers seeking to allocate resources (e.g., cancer care, prevention and research) and to assess the economic burden of cancer. The future number of new cancer cases will depend on three main components. The first is an epidemiological component, i.e. the change in age-standardized cancer incidence rates, which approximates the change in individual risk factors as well as in preventive actions taken to counter them [1]. The other two components are demographic, i.e. the growth and aging of the population observed in many countries.

In general, prediction of the future number of cancer cases in a country is made by combining a prediction of age-standardized cancer incidence rates, obtained by models that attempt to identify and extend past trends into the future, with demographic projections of population size and age structure [2]. Methods to predict age-standardized cancer incidence rates most often used Age-Period-Cohort (APC) models, considering these three dimensions as proxies for different risks and preventive factors [2–5]. More recently, methods such as Bayesian Age-Period-Cohort (BAPC) models [6, 7], joinpoint regression [8, 9] and machine learning [10] have also been used. The choice of the statistical method can, however, have a significant impact on prediction. A recent study comparing a large set of prediction models showed the superiority of ARIMA (Auto Regressive Integrated Moving Average) methods which consistently outperformed more sophisticated methods [11].

In Switzerland, the National Agency for Cancer Registration (NACR) gathers data from regional cancer registries and produces national incidence figures, extrapolating the expected number of cancers for the few areas not covered by population-based registries [12, 13]. A study used these data for the period 1989–2009 to predict, by APC modelling, the evolution of age-standardized incidence rates up to the period 2015–19 for all cancers combined and for each of the 12 most frequent cancer sites [14]. Predicted age-standardized cancer incidence rates were then combined with population projections from the Swiss Federal Statistical Office (FSO) to forecast the number of new cancer cases to 2015–19. Eight years later, with Swiss cancer incidence series available up to the year 2018, these predictions can be compared with the observed trends, while new predictions are needed.

The first objective of the present study was to predict the age-standardized cancer incidence rates and the absolute number of new cancer cases in Switzerland, for all cancers combined and for each of the 12 most frequent cancers from 2019 until 2025. This was done combining NACR incidence data and Swiss population demographics projections. For the statistical analysis, we used a low order Auto Regressive Integrated Moving Average (ARIMA) model [15] for predicting age-standardized cancer incidence rates, as recommended by [11], while adopting the FSO projections for the demographic evolution. A second objective was to quantify the contribution of the change in the individual cancer risk, as approximated by age-standardized cancer incidence rates, and of population aging and growth to the expected change in the number of new cancer cases between 2018 and 2025.

## Method

### Cancer incidence data

As primary source of data, we used cancer incidence data that were calculated and produced by the Swiss National Agency for Cancer Registration (NACR) for all Switzerland. For this, NACR gathered data recorded by regional population-based cancer registries in Switzerland and extrapolated national figures including regions not covered by a registry. NACR made available for years y = 1987–2018 the age standardized cancer rates $\lambda_{\text{y}}^{\text{s}}$ at the European standard population *s* [16]:

$$\lambda_{y}^{s}=\sum_{a\in A}\lambda_{y,a}P_{s,a}/P_{s}$$ (1)

In (1), *A* is a partition of the ages into 5-year age groups (i.e. 0–4, 5–9, …, last open group 85 years and over), λ_y,a_ is the age-specific incidence rate for the year y and age group *a* (*a* ∈ *A*) (e.g. *a* = 0–4 years), and P_s,a_/P_s_ is the proportion of people in the age group *a* in the standard population *s*. We considered age-standardized cancer incidence rates for the 12 most frequent cancer sites according to the ICD10 codes: oral cavity and pharynx (C00-14), stomach (C16), colon and rectum (C18-20), lung-bronchus-trachea (C33-34), skin melanoma (C43), breast (C50), corpus uteri and uterus NOS (C54-55), ovary (C56), prostate (C61), bladder (C67), thyroid (C73), non-Hodgkin lymphoma (C82-85, C96). All other cancers were regrouped in the category “other” (All other ICD10 codes except C44) to allow calculation of the overall cancer incidence (C00-43, C45-97).

### Demographic data

Our projections of cancer incidence based on NACR data were combined with demographic projections for the Swiss population. For the evolution of the Swiss population from 2021 up to 2025, we used the so-called “average A00-2020” reference scenario of the Swiss Federal Statistical Office (FSO) [17]. It predicts globally and by age groups the most plausible growth of the population based on demographic changes in mortality, migration, and fertility. This reference scenario predicts a number of women increasing from 4,292,551 in 2018 to 4,538,813 in 2025 (+5.74%) and a number of men increasing from 4,221,778 in 2018 to 4,482,174 in 2025 (+6.17%). For years up to 2020, we used the official population numbers from the FSO.

### Modelling and prediction of age-standardized cancer incidence rates

For the choice of a statistical method to predict age-standardized cancer incidence rates, we referred to a recent comparison of the accuracy of several models and methods, based on the repeated application of leave-future-out cross-validation on a 35-year series of incidence data from cancer registries [11]. According to [11], the best predictions are obtained by simply extrapolating trends in standardized cancer rates (1) by ARIMA (Auto Regressive Integrated Moving Average, [15]) models. These models showed better accuracy than the more complex and widely used APC methods and have the advantage of requiring only the age-standardized rates, while the age-specific incidence rates are not needed. The simple ARIMA(2,1,1) proved to be the best performing method and was adopted for the present study. Mathematically, this model can be described as follows. ARIMA(2,1,1) combines a differenced 2-order autoregressive model with a 1-order moving average model, which can be written as:

$$d\lambda_{y}^{s}=c+\alpha_{1}d\lambda_{y-1}^{s}+\alpha_{2}d\lambda_{y-2}^{s}+\epsilon_{y}+\theta_{1}\epsilon_{y-1}$$ (2)

In (2), ${\text{d}\lambda }_{\text{y}}^{\text{s}}$ (y = 1987–2018) are differences between age-standardized rates at two consecutive years, ${\text{d}\lambda }_{\text{y}}^{\text{s}}=\lambda_{\text{y}}^{\text{s}}-\lambda_{\text{y}-1}^{\text{s}}$, ∈_y_ are normally distributed random errors, and *c*, *α*_1_, *α*_2_, θ_1_ are a constant, two auto-regressive coefficients and one moving average coefficient, respectively. This model has been fitted to the NACR data described above. Coefficients in model (2) were estimated separately for each cancer site and for each sex, when applicable. We used the ARIMA function from software R [18] to estimate these coefficients, to estimate (with 95% prediction intervals) age-standardized cancer incidence rates ${\hat{\lambda }}_{\text{y}}^{\text{s}}$ for years y = 1987–2018, and to predict them (with 95% prediction intervals) for years y = 2019–2025. Technically, the estimation method implemented in R is based on [19].

The percent change in age-standardized cancer incidence rates between 2018 and 2025 was then estimated by comparing the predicted value for 2025, ${\hat{\lambda }}_{2025}^{\text{s}}$, with estimated value for 2018, ${\hat{\lambda }}_{2018}^{\text{s}}$, instead of the observed value $\lambda_{2018}^{\text{s}}$. This choice was intended to reduce the impact of random fluctuations in 2018, ∈_2018_, on our comparisons.

### Prediction of the future number of new cancer cases

In order to predict the absolute number of new cases expected each year ${\hat{\text{N}}}_{\text{y}}$, y = 2019–2025, the predicted age-standardized rates ${\hat{\lambda }}_{\text{y}}^{\text{s}}$ should be conveniently combined with the FSO population forecasts by 5-year age classes for the same years. We used the following approximation:

$${\hat{N}}_{y}\approx P_{y}∙{\hat{\lambda }}_{y}^{s}∙\lambda_{y^{⋆}\;}^{y}/\lambda_{y^{⋆}\;}^{s}$$ (3)

Here, y⋆ is any year in the period 1987–2018 (we considered y⋆ = 2018); y is a future year for which we wish to make the prediction (y = 2019–2025); *P*_*y*_ is the total population for year y according to the FSO forecast [17]; ${\hat{\lambda }}_{\text{y}}^{\text{s}}$ are predicted standardized rates for year y according to model (2); $\lambda_{y^{⋆}\;}^{y}=\sum_{a\in A}\lambda_{y^{⋆},a}P_{y,a}/P_{y}$ (NACR) are cancer rates of year y⋆ standardized on the population structure at future year y (FSO forecast); and $\lambda_{\text{y}*}^{\text{s}}$ are cancer rates in year y⋆ standardized on the European standard population s. Approximation (3) is valid provided that the rate ratio between any two age groups a_1_, a_2_ ∈ A remains approximately stable over time, that is: $\lambda_{y,a_{1}}/\lambda_{y,a_{2}}\approx \lambda_{y^{⋆},a_{1}}/\lambda_{y^{⋆},a_{2}}$; y ∈ 2019–2025, y* = 2018. In that case, the ratio of standardized rates for years y and y* will not be much affected by the particular choice of standardization, so that ${\hat{\lambda }}_{\text{y}}^{\text{s}}/\lambda_{\text{y}⋆}^{\text{s}}\approx {\hat{\lambda }}_{\text{y}}^{\text{y}}/\lambda_{\text{y}⋆}^{\text{y}}$, ensuring validity of (3). Empirically this is generally the case, as shown by [20].

### Factorization of the predicted change in number of new cancer cases

The predicted change in the number of new cancer cases between 2018 and 2025 was factorized into three components [21]. Considering that $N_{2018}=P_{2018}\lambda_{2018}^{2018}$, the change (in %) of the number of new cancer cases between 2018 and 2025 can be expressed as:

$$D=100∙\left[{\hat{N}}_{2025}/N_{2018}-1\right]\%=100∙\left[(1+A/100)∙(1+B/100)∙(1+C/100)-1\right]\%$$ (4)

In (4), $A=100({\hat{\lambda }}_{2025}^{s}/\lambda_{2018}^{s}-1$) is the change (in %) in the number of new cancer cases due to the change in individual cancer risk, as approximated by age-standardized cancer rates, $B={100(\lambda }_{2018}^{2025}/\lambda_{2018}^{2018}-1)$ is the change (in %) in the number of new cancer cases due to the population ageing, and *C* = 100(*P*_2025_/*P*_2018_ − 1) the change (in %) due to the population growth. This factorization allows us to quantify the multiplicative contributions of the epidemiological component (A) and the two demographic components (B and C) on the expected future number of new cancer cases (D).

## Results

**Fig 1:**
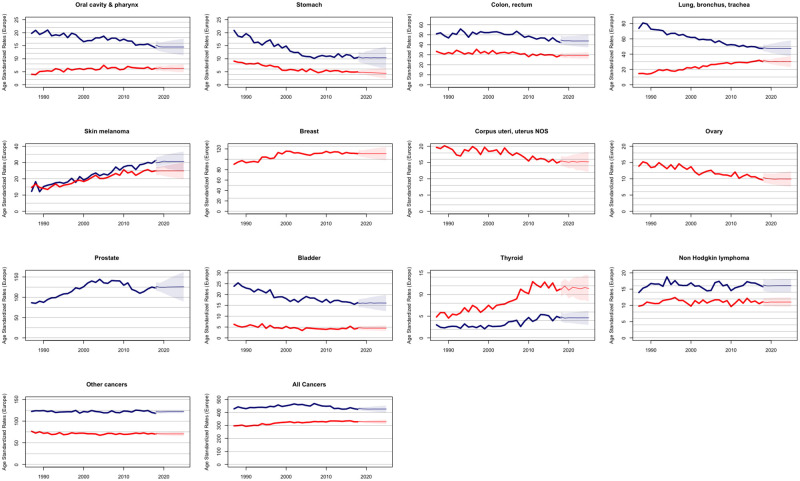
Observed and projected age standardized rates for all cancers and 12 cancer sites in Switzerland. Bold lines represent actual rates; thinner lines represent projected rates; shaded areas represent 95% prediction intervals. Men in blue; women in red.

**Fig 2:**
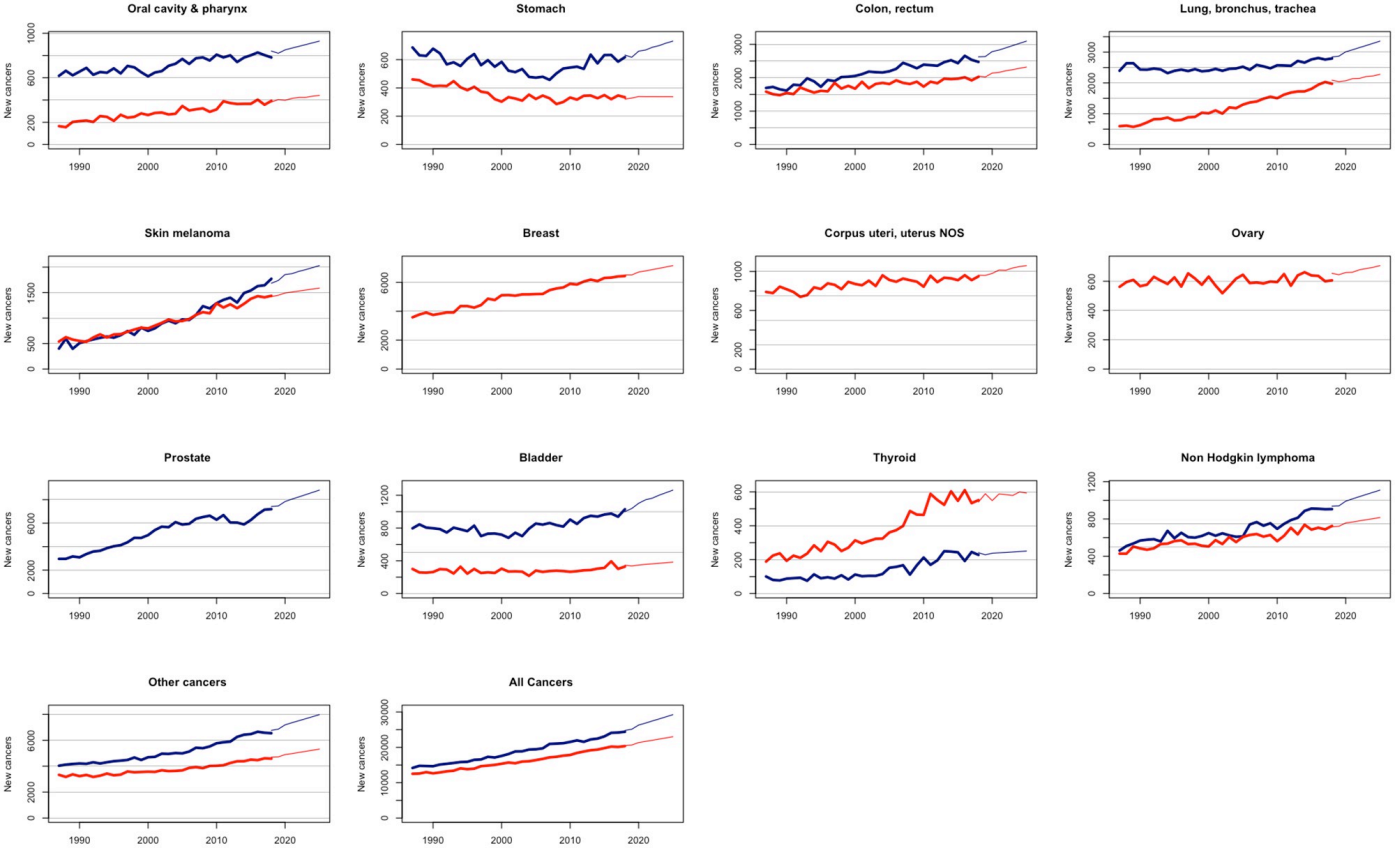
Observed and projected numbers of new cancer cases for all cancers and 12 cancer sites in Switzerland. Bold lines represent actual numbers; thinner lines represent projected numbers. Men in blue; women in red.

Over the past decade, we have observed in Switzerland a stabilization of age-standardized cancer incidence rates and a steady increase in the number of new cancer cases, as previously predicted [14]. Our new predictions for age-standardized cancer incidence rates and absolute number of cancer cases up to 2025 point to a continuation of these trends (Figs 1 and 2). While age-standardized rates are expected to remain roughly stable in the coming years (Fig 1), the number of cancer cases is predicted to increase substantially for each cancer site and for all cancers combined (Fig 2).

Table 1 shows the expected change in the estimated number of new cancer cases between 2018 and 2025 for each cancer site and sex. This change is also factorized into its epidemiological and demographic components, as explained in the Method section, Eq (4). The age-standardized cancer incidence rates, reflecting changes in cancer risk, are overall expected to remain roughly stable until 2025 for both sexes (see also Fig 1). For men, a slight increase in incidence rate is expected for cutaneous melanoma (+2.75%), and bladder cancer (+1.24%), whereas the incidence rates are expected to decrease slightly for cancers of the lung, bronchus and trachea (-1.38%), oral cavity and pharynx (-4.61%), stomach (-3.72%), colon-rectum (-2.15%), thyroid (-5.07%), and for non-Hodgkin’s lymphoma (-1.22%) and to remain approximately constant for prostatic cancer (-0.66%). For women, a slight increase in standardized incidence rate is foreseen for cancers of the oral cavity and pharynx (+1.99%) and thyroid (+2.91%), while a slight decrease is predicted for cancers of the colon-rectum (-1.98%), lung, bronchus and trachea (-4.86%), breast (-1.08%), uterus (-2.24%), ovary (-4.17%) and bladder (-5.09%), and more markedly for stomach cancer (-8.13%). No change in incidence rate is expected for cutaneous melanoma (+0.68%) and non-Hodgkin’s lymphoma (+0.17%) in Swiss women.

**Table 1:** Estimated numbers of cancer cases for 2018 and predictions for 2025 in Switzerland by cancer site and by sex. Overall predicted changes are factorized (multiplicatively) into changes in standardized risk (A), in population structure (B), and in population size (C) (Eq (4)).

	MEN						WOMEN					
CANCER SITE	Number of cases 2018	Number of cases 2025	Change in risk A (%)	Change in pop structure B (%)	Change in pop size C (%)	Overall change (%)	Number of cases 2018	Number of cases 2025	Change in risk A (%)	Change in pop structure B (%)	Change in pop size C (%)	Overall change (%)
**ORAL CAVITY & PHARYNX**	841	929	-4.61	9.07	6.17	18.46	382	442	1.99	7.29	5.74	15.71
**STOMACH**	632	731	-3.72	13.15	6.17	15.66	321	338	-8.13	8.4	5.74	5.3
**COLON, RECTUM**	2625	3095	-2.15	13.49	6.17	17.9	2050	2319	-1.98	9.14	5.74	13.12
**LUNG, BRONCHUS, TRACHEA**	2844	3358	-1.38	12.76	6.17	18.07	2088	2280	-4.86	8.55	5.74	9.2
**SKIN MELANOMA**	1684	2029	2.75	10.45	6.17	20.49	1421	1589	0.68	5.04	5.74	11.82
**BREAST**							6515	7156	-1.08	5.01	5.74	9.84
**CORPUS UTERI, UTERUS NOS**							961	1060	-2.24	6.7	5.74	10.3
**OVARY**							654	706	-4.17	6.53	5.74	7.95
**PROSTATE**	7420	8815	-0.66	12.64	6.17	18.8						
**BLADDER**	1000	1262	1.24	17.41	6.17	26.2	346	383	-5.09	10.3	5.74	10.69
**THYROID**	241	251	-5.07	3.34	6.17	4.15	540	593	2.91	0.91	5.74	9.81
**NON HODGKIN LYMPHOMA**	937	1109	-1.22	12.85	6.17	18.36	716	815	0.17	7.47	5.74	13.83
**OTHER CANCERS**	6763	7974	0.07	10.98	6.17	17.91	4695	5318	-1.31	8.54	5.74	13.27
**ALL CANCERS**	24987	29553	-0.89	12.40	6.17	18.27	20689	22999	-0.78	5.96	5.74	11.17

The total number of new cancer cases is expected to increase between 2018 and 2025 from 24,987 to 29,553 (+18%) for men and from 20,689 to 22,999 (+11%) for women, i.e. an absolute increase of 6,876 new cancer cases (+15%) for both sexes. This increase is almost entirely attributable to the projected aging (+12% for men and +6% for women) and growth (+6% for both sexes) of the Swiss population, since the contribution of the evolution of cancer risk amounts to less than 1% for both sexes. A rise in the number of new cancer cases is predicted for each cancer site and sex, with increases ranging from 4.15% for thyroid in men to 26% for bladder in men. Here also, all increases are primarily due to the demographic evolution, with the contribution of population aging generally outweighing that of population growth, with the notable exception of thyroid cancer for both sexes (Table 1). Only for thyroid cancer in women and cutaneous melanoma in men, an increase in risk partly contributed to the overall increase in the expected number of new cancer cases. Of note, for the few sites where we forecast a decrease in cancer risk (for example stomach cancer in women), this decrease is insufficient to offset the increase due to population growth and aging.

## Discussion

In this study, we presented projections of the burden of cancer in Switzerland until 2025, in terms of both age-standardized rates and number of new cases, using, for the first time, the simple ARIMA method. Altogether, cancer incidence is predicted to stabilize for men and women, and for most cancer sites. The number of cases is, however, expected to increase by 15% (from 45,676 in 2018 to 52,552 in 2025), more so in absolute and relative terms for men than women. This increase is almost entirely driven by demographic changes. For men, it is primarily due to the projected population aging (+12.4%) followed by population growth (+6.2%) whereas the contributions of population aging and growth to the increase were comparable for women (6.0% and 5.7%, respectively).

For all cancers combined, the previous predictions until 2015–19 [14] closely corresponded to the observed Swiss figures until 2018 [22], i.e. a stable incidence rate for women and a slightly decreasing incidence rate for men, with an increasing number of cancers for both sexes. For a few sites such as stomach cancer in men and breast cancer in women, we observed, however, a less marked dynamic than was expected. This means a lesser decrease in incidence rate and therefore a larger increase in the number of cancers than predicted by [14]. For breast cancer, the implementation of mammography screening programs in several Swiss cantons since 2010, leading to the earlier detection of cancers that would otherwise be diagnosed a few years later, may explain the difference between predicted and observed breast cancer incidence rates [23].

Using the ARIMA method recommended by a large comparison study [11], we forecast a continuation until 2025 of the recent stable trends in age-standardized incidence rates for all cancers combined (around 426/100,000 in men and 328/100,000 in women) and a stabilization of incidence for most cancer sites considered. A stabilized age-standardized incidence can result from the continuation of past recent trends (colon-rectum, breast, prostate, bladder and non-Hodgkin lymphoma), the leveling off of a downward trend (for men: oral cavity, pharynx, lung and stomach; for women: corpus uteri, uterus, ovary and thyroid) or the leveling off of an upward trend (cutaneous melanoma for both sexes and oral cavity, pharynx and lung for women). While no substantial increase in incidence is forecast for any cancer site, the incidence of stomach cancer in women is predicted to substantially decrease (-8.13%), continuing the recently reported downward trend in Switzerland [22]. Similarly, the incidence of thyroid cancer in men, which recorded the greatest increase in incidence of all cancer sites in Swiss men between 2008–2012 and 2013–2017 [22], is predicted to materially decrease (-5.07%) up to 2025.

Interpretation of cancer projection is challenging. The latency period between preventive measures aiming at reducing exposure to specific risk factors and its resulting effect on cancer incidence–and its magnitude–are difficult to anticipate. Further, the effect of early detection activities is often difficult to account for. This is particularly true for Switzerland where initiation of organized cancer screening programs and early detection activities mostly occur at regional level, without uniform or simultaneous application at the national level, and changes in clinical and diagnostic practices are virtually unpredictable. However, predicted incidence trends for several cancers are nevertheless largely corroborated by the Swiss epidemiological context. For example, the expected decrease, or stabilization after a long period of decrease, for males in incidence of cancers largely attributable to tobacco (oral cavity & pharynx, lung, bladder) and the predicted stabilization of incidence for those tobacco-associated cancers in females, after a long period of increase, are in line with the reduction of smoking prevalence and smoking ban policy in public places, which occurred later in Switzerland than in most other countries. The plateauing incidence rate of prostate cancer, the most common cancer in Swiss men, after years of a steady increase followed by a decrease has largely been attributable to changes in PSA screening and clinical workup practices [24]. Analogously, the strong rise between 1998 and 2012 in incidence of thyroid cancer in Switzerland, more pronounced in women than in men, was shown to be limited to small papillary carcinoma and likely due to overdiagnosis [25, 26]. The predicted stabilization of incidence in women and slight decrease in men may reflect recent favourable changes in clinical and diagnostic practices, as for prostate cancer. The predicted attenuation of the rise in melanoma incidence, after years of steady increase, has also been observed in countries with longstanding and sustained prevention activities against skin cancer [27–29]

In terms of predicted number of future new cases by 2025, the ranking of the three most common cancers will remain unchanged in the coming years for men (1^st^ prostate, 2^nd^ lung, 3^rd^ colon-rectum), but colorectal cancer is expected to overtake by 2025 lung cancer as the second most frequent female cancer in Switzerland, behind breast cancer.

Comparing our results with recent projection studies from other industrialized countries, we note that a similar stabilization of incidence rates has overall been expected in 2020 (using data from 2010–12) in a study applying machine learning algorithms to predict incidence rates of all cancers and of the four main cancer types (lung, breast, prostate and colon) in Europe [10]. A study applying a BAPC model recently predicted for Australia an annual increase of 3% in the number of cancer cases between 2016 and 2031 [7]. This annual increase corresponds to an increase of 100(1.03^7^ − 1)% = 23% over a 7-year period, which is higher than the 15% increase obtained in Switzerland over the 7-year period 2018–2025. This difference may partly be explained by a more marked dynamic of the age-standardized incidence rates predicted for Australia, i.e. a slight decrease for colon, stomach, and lung cancer in men and an increase for melanoma and lung cancer in women, in contrast to the stabilization expected for Switzerland. However, direct comparisons between countries should consider differences in exposure to risk factors, preventive measures, screening and intensity of diagnostic procedures, even if the evolution of the demographic components appears similar in Australia and Switzerland, with a continuous growth of life expectancy [30]. In this respect, the situation differs from the United States, where life expectancy has been stagnating or even decreasing in the past ten years. As demographic factors strongly influence the future number of cancers, this could explain, at least in part, the recent prediction of an overall relatively small rise from 1,735,000 new cancer cases in 2020 to 1,881,000 in 2040 in the United States [9], contrasting with our projections for Switzerland and those for Europe [10] and Australia [7].

This study has strengths and inherent limitations. One strength is the adoption of the ARIMA method for prediction. In addition to its demonstrated validity in a model comparison study based on a large number of leave-future-out cross-validation scenarios [11], this method can be directly applicable to standardized rate series without requiring ad hoc methods to mitigate the predicted dynamics (dumping or forced stabilization), as is often the case for APC models [5, 14]. However, as the ARIMA models predict standardized rates but not age-specific rates, an approximation [20] was necessary to combine these predictions with the demographic forecasts to obtain predicted numbers of cancer cases. A second strength of this study is that it uses annual rates rather than 5-year average rates for prediction, as is the more usual practice [5, 14]. Our approach allows us to use more detailed information and to predict future trends more accurately. However, this choice also entails a limitation when we want to quantify the variation of the rate between an observed year (2018) and a future year (2025), regarding the random fluctuations that may occur in a specific year (the year 2018 may present a rate slightly higher or lower than the trend, precisely due to this random variability). To mitigate this effect, we decided to compare the prediction for 2025 to the rate estimated by the model for 2018, rather than to the observed rate. As ARIMA models are based on moving averages, this choice smoothed out random fluctuations.

The major limitation of this study is the implicit assumption that no disruptive or unpredictable event will interfere with the continuation of past trends. This assumption, inherent to any prediction of the future from the past, has been violated twice. First, the Swiss cancer registration system was profoundly reformed in 2020 when notifications of all oncological diseases became mandatory. While the Cancer Registration Act (CRA) enabled full coverage of the population, constraints on patients informed consent and rights to veto prior to any case registration should affect the future completeness of registration to an—as yet—unknown degree [31, 32]. The magnitude of this disruption appears very likely to exceed any inaccuracy in the number of registered cancers (exhaustivity generally exceeds 95% in Swiss registries for most cancer sites [12]) or any imprecision in the extrapolation method used by NACR for regions not covered by cancer registry between 1987 and 2018. In this respect, our projections will provide a valuable baseline to estimate the effect of the CRA. Second, the unexpected COVID-19 pandemic produced some disruption in demographics in the last two years due to an increased mortality, particularly in older population groups where cancer risk is highest. Whether the reference scenario of the Swiss Federal Office for Statistics for population projection we used still holds true is unknown. Delayed cancer diagnosis due to the pandemic and the lockdown could, to some extent, also temporarily reduce cancer incidence and differently for various cancer types [33]. No model applied to pre-pandemic data could anticipate this kind of evolution. For these reasons, we believe that any prediction should not go too far into the future. We have limited ourselves to 7 years. Another limitation is the time lag inherent to complete cancer registration data which meant that, as of 2022, 2018 was the latest available year for predicting the Swiss cancer burden until 2025. The final limitation, which our study shares with all studies combining a prediction of incidence rates with population projections, is the difficulty to obtain prediction intervals for the absolute numbers of cancer cases as the uncertainty associated with the prediction of population figures is not available. This could be the subject of future work.

Despite these limitations, we can reasonably conclude that our simple prediction method based on ARIMA model was able to project trends in cancer incidence rate and absolute numbers of cancers until 2025 which overall concurred with the epidemiological context and knowledge in Switzerland. These trends generally pointed towards a stabilization of age-standardized incidence rates for most cancers, accompanied by a substantial increase in the numbers of all types of cancer, driven by demographic changes. To counter the unavoidable increase in cancer cases, more effective and sustained prevention measures targeting factors such as obesity, physical activity, healthy diet and tobacco use are necessary. Structural interventions should also be devised in order to prevent any potential shortage of professionals in cancer care delivery.
